# A systematic review comparing the macrophage inflammatory response to hydrophobic and hydrophilic sandblasted large grit, acid‐etched titanium or titanium–zirconium surfaces during in vitro studies

**DOI:** 10.1002/cre2.730

**Published:** 2023-03-29

**Authors:** Eamonn Donohoe, Rawan Kahatab, Fadi Barrak

**Affiliations:** ^1^ School of Dentistry University of Central Lancashire Preston UK; ^2^ Department of Oral and Maxillofacial Surgery University Hospital Galway Galway Ireland

**Keywords:** inflammation, macrophages, surface properties, titanium

## Abstract

**Objectives:**

Macrophages are among the first cells to interact with the dental implant surface and are critical regulators for controlling the immune response toward biomaterials. Macrophages can polarize between two main phenotypes: proinflammatory M1 macrophages and anti‐inflammatory M2 macrophages. This systematic review aims to determine if a differing macrophage inflammatory response exists on hydrophilic sandblasted large grit, acid‐etched (SLActive) surfaces compared to sandblasted large grit, acid‐etched (SLA) titanium or titanium–zirconium surfaces during in vitro studies.

**Material and Methods:**

A systematic search of three electronic databases, Medline, DOSS (Dentistry and Oral Sciences Source), and WoS (Web of Science), was performed. Only in vitro studies were included in this systematic review. The electronic search was supplemented with a search of the references. Genetic expression and production of proinflammatory and anti‐inflammatory proteins were assessed. The synthesis of quantitative data was completed by narrative synthesis.

**Results:**

A total of 906 studies were found with the systematic search. Eight studies remained after the application of inclusion and exclusion criteria. Six studies used murine macrophages, while two used human macrophages. Discs were used in six studies, while dental implants were used in the remaining two studies. Genetic expression and cytokine production of proinflammatory cytokines on SLActive surfaces were reduced compared to SLA. Anti‐inflammatory genetic expression and cytokine production was increased on SLActive surfaces. The overall quality of the included studies was low to moderate.

**Conclusions:**

SLActive surfaces modulate macrophages to reduce proinflammatory and increase anti‐inflammatory gene expression and cytokine production compared to SLA surfaces. The in vitro nature of the included studies does not replicate the in vivo healing cascade. Further in vivo studies are required to assess the macrophage response toward SLActive implant surfaces compared to SLA surfaces.

## 1 INTRODUCTION

Osseointegration is defined as “a direct structural and functional connection between ordered, living bone and the surface of a load‐bearing implant” (Listgarten et al., 1991). After implant insertion into the bone, a rapid protein adsorption from the patient's blood on the implant's surface occurs. These proteins include fibronectin, fibrinogen, albumin, immunoglobulins, and complement C3. This stage is followed by nonspecific cellular adhesion, which occurs through the extracellular matrix interface (Batool et al., 2021; Mariani et al., 2019). The osseointegration of dental implants relies upon the cell's reaction to surface modifications, which is related to this protein adsorption (Trindade et al., 2015). Bone formation around implants can be via contact osteogenesis or distance osteogenesis (Davies, 2003). The initial inflammatory immune response is critical for bone formation, osseointegration, and successful regenerative capacity and macrophages are widely involved (Oishi & Manabe, 2018).

Macrophages are among the first cells to interact with the dental implant surface (Bosshardt & Pippenger, 2021). These immune cells are critical regulators for controlling the immune response toward biomaterials, controlling the inflammatory response and orchestrating the healing process (Krzyszczyk et al., 2018; Lee & Bance, 2019). Macrophages can polarize into two main phenotypes: the antimicrobial and proinflammatory M1 macrophages and the anti‐inflammatory and proregenerative M2 macrophages (Italiani & Boraschi, 2014). M1 macrophages can release proinflammatory cytokines, including interleukin‐1β (IL‐1β), IL‐6, IL‐12, and tumor necrosis factor‐α (TNF‐α) (Sica & Mantovani, 2012). In contrast, M2 macrophages express CD163 and produce IL‐10 (Komohara et al., 2006; Martinez et al., 2009). IL‐4 has also been shown to be made by M2 macrophages (La Flamme et al., 2012).

Macrophages appear to orchestrate the inflammatory response toward biomaterials and tissue damage (Sheikh et al., 2015). A healing environment with an unbalanced M1/M2 macrophage ratio with a dominant M1 macrophage phenotype may lead to low‐grade inflammation, osteolysis, and loosening of implants (Goodman et al., 2014). In addition, prolonged unresolved inflammation can lead to the fusion of macrophages into foreign body giant cells (Wynn & Barron, 2010). Furthermore, this inflammatory process may lead to fibroblast recruitment and bring about a fibrous encapsulation and failure of osseointegration (Wynn & Barron, 2010). Modulation of the macrophage phenotype has been found to be critical in osseointegration (Trindade et al., 2018a). A balanced M1/M2 macrophage ratio has been related to M2 macrophage‐linked bone growth after 10 days of healing at the peri‐implant site (Trindade et al., 2018b). In addition, it has been shown that M2 macrophages contribute to the ossification phase of fracture repair (Schlundt et al., 2018).

Implant surface modifications attempt to improve primary implant stability, accelerate osseointegration, and maintain osseointegration (Bosshardt & Pippenger, 2021). In addition, this may allow for a decreased healing time and an earlier loading of the implant fixture (Buser et al., 2017). Implant surface modifications can be through mechanical (e.g., grit‐blasting), chemical (e.g., acid or alkali treatments), or physical means (e.g., plasma spraying) (Barfeie et al., 2015). In addition, chemical modification can increase dental implants' surface energy and hydrophilic properties (Bosshardt & Pippenger, 2021).

Straumann dental implants are composed of either grade IV titanium or titanium–zirconium alloy, otherwise known as Roxolid® (Bosshardt & Pippenger, 2021). Roxolid® has been shown to have a 10%–15% higher ultimate tensile strength and enhanced fatigue performance compared to grade IV titanium (Medvedev et al., 2016). Straumann implants may have a hydrophobic sandblasted large grit, acid‐etched (SLA) surface or a hydrophilic sandblasted large grit, acid‐etched (SLActive) surface, also known as modified SLA (modSLA) (Bosshardt & Pippenger, 2021). SLA implants are dry and stored after sandblasting and acid etching. In contrast, SLActive implants are rinsed under protective N2 gas conditions, rinsed with isotonic water, and stored in a saline solution (Rupp et al., 2006). This process results in a hydrophilic surface with higher surface energy and less hydrocarbon contamination from the atmosphere (Schwarz et al., 2009).

Currently, there is a lack of evidence in the literature regarding the immunological response toward modified implant surfaces (Bosshardt & Pippenger, 2021). There are no systematic reviews investigating the macrophage inflammatory response towards SLA and SLActive surface modifications on titanium or titanium–zirconium.

This systematic review was used to determine if a differing macrophage inflammatory response exists for SLActive surfaces when compared to hydrophobic SLA surfaces on titanium or titanium–zirconium during in vitro studies.

### 1.1 Review question

Is there a difference in macrophage inflammatory response to hydrophobic SLA compared to SLActive titanium or titanium–zirconium surfaces during in vitro studies?

The review question was formulated using participants, interventions, outcomes, and study design (PICOS) framework (Centre for Reviews and Dissemination, 2008), outlined in Table 1.

**Table 1:** PICOS framework.

Participants	Macrophages are placed on the surface of the substrate.
Intervention	SLActive commercially pure titanium (grades I–IV) or titanium–zirconium alloy
Comparison	Hydrophobic SLA commercially pure titanium (grades I–IV) or titanium–zirconium alloy
Outcomes	The inflammatory response of macrophages on the surface substrate will include:
	Cytokine production Genetic expression of inflammatory cytokine fold change Expression of macrophage surface markers
Study design	In vitro studies

Abbreviations: PICOS, participants, interventions, outcomes, and study design; SLA, sandblasted large grit, acid‐etched.

## 2 MATERIALS AND METHODS

A systematic search of Search terms along with Medical Subject Headings (MeSH) was used. When the database did not support the use of MeSH, these terms were searched as keywords. Truncation was used where appropriate. The Boolean operators “OR” and “AND” were used. The Boolean operator “NOT” was not used to avoid the exclusion of potentially relevant studies. Three electronic databases were used: Medline, Dentistry and Oral Sciences Source (DOSS) and Web of Science (WoS). These databases were searched on February 2, 2022.

While completing the searches, no language restrictions, date limits, or search filters were applied.

The inclusion criteria for studies were as follows: (1) *Participants*: Macrophages from murine or human sources were included. (2) *Intervention*: Studies which placed macrophages on SLActive titanium or titanium–zirconium surfaces. (3) *Comparison*: Studies in which the placement of macrophages on hydrophobic SLA titanium or titanium–zirconium surface is undertaken. The intervention and the comparator materials will be composed of commercially pure titanium (grades I–IV) or titanium–zirconium. (4) *Outcomes measures*: Genetic expression of proinflammatory and anti‐inflammatory cytokines, the evaluation of proinflammatory and anti‐inflammatory cytokines production and the presence of anti‐inflammatory macrophage surface markers. Table 2 outlines the proinflammatory and anti‐inflammatory markers used for the tested macrophages. (5) *Study design*: In vitro studies will be included to allow results to be compared due to relatively homogeneous study methodologies. When a study has an in vitro and in vivo component, the sections relevant to the in vitro sections of the study will be included in this systematic review.

**Table 2:** Outline of proinflammatory and anti‐inflammatory outcome markers that will be used in determining the inflammatory response of macrophages on SLA and SLActive surfaces.

Proinflammatory	Anti‐inflammatory
IL‐1β	IL‐4
IL‐6	IL‐10
IL‐12	CD163
TNF‐α	

Abbreviations: IL, interleukin; SLA, sandblasted large grit, acid‐etched; SLActive, hydrophilic sandblasted large grit, acid‐etched; TNF, tumor necrosis factor‐α.

Excluded studies included those which had polarized macrophages before their placement onto the surface substrate and studies with intermediate cells, substrates, or proteins present on the test surfaces before the seeding of the macrophages (e.g., platelet proteins). No additional limits will be used (e.g., language or publication type).

The “Template for assessing the quality of simple lab studies” is a quality and risk of bias tool, which had been developed at the University of Central Lancashire and was provided by N. C. as shown in Supporting Information: Appendix 1.

A narrative synthesis was conducted for the data synthesis approach in this systematic review (Campbell et al., 2018).

## 3 RESULTS

**Figure 1:**
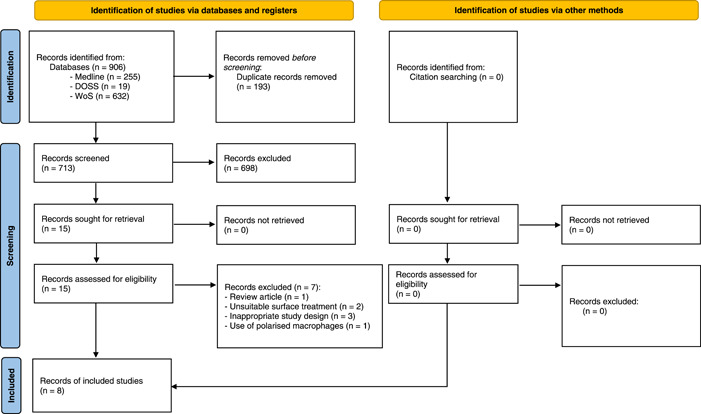
PRISMA flowchart. DOSS, Dentistry and Oral Sciences Source; WoS, Web of Science.

A total of 906 studies were identified. A full search history for each database can be found in Supporting Information: Appendix 2. Eight studies met the eligibility criteria of this systematic review. A search of the references of the included studies was performed, and zero further studies were for inclusion. We excluded seven studies that are outlined in Supporting Information: Appendix 3. A PRISMA flow diagram representing the above results from the review process is depicted in Figure 1.

### 3.1 Characteristics of included studies

Of the eight studies that met the eligibility criteria, six were in vitro studies, and a further two studies had an in vitro and an in vivo component (Abaricia et al., 2021; Hotchkiss et al., 2018). Six studies used macrophages from a murine source, with two studies of these using a RAW 264.7 cell line (Hamlet et al., 2012; Wang et al., 2019). The four other studies used macrophages derived from C57BL/6 mice (Hotchkiss et al., 2016, 2017, 2018, 2019). In addition, macrophages from human studies were used in two studies, with one using a human acute monocytic leukemia cell‐line THP‐1 (Alfarsi et al., 2014) and the other study derived their macrophages from human peripheral blood monocytes (Abaricia et al., 2021).

Six studies had the materials in a disc shape, measuring 1 mm in thickness and 15 mm in diameter, with four studies using titanium discs only (Alfarsi et al., 2014; Hamlet et al., 2012; Hotchkiss et al., 2016, 2018). The other two studies used titanium and titanium–zirconium discs (Hotchkiss et al., 2017; Wang et al., 2019). Two studies used dental implants, of which one study used titanium–zirconium implants (Hotchkiss et al., 2019), while the other study used titanium and titanium–zirconium implants (Abaricia et al., 2021).

All studies had three technical replicates. In addition, four studies repeated experiments three times (Hamlet et al., 2012; Hotchkiss et al., 2017, 2019; Wang et al., 2019). Abaricia et al. (2021) and Hotchkiss et al. (2016) repeated experiments at least twice. In contrast, it was not clearly reported that any repeat experiments were performed by Alfarsi et al. (2014) and Hotchkiss et al. (2017).

Full tables of study characteristics and findings are presented in the Supporting Information: Appendix 4.

### 3.2 Findings

#### 3.2.1 Gene expression

*At 24 h*: Hamlet et al. (2012) found that titanium discs with a hydrophilic SLA surface significantly (*p* < .05) downregulated gene fold expression of IL‐1β (−11.91) and TNF‐α (−1.86) in comparison to hydrophobic SLA. Similarly, Hotchkiss et al. (2019) found that IL‐1β, IL‐6, and TNF‐α were significantly downregulated on hydrophilic SLA surfaces on titanium–zirconium dental implants when compared to SLA surface treatment. Hotchkiss et al. (2017) found that IL‐1β, IL‐6, and TNF‐α gene expressions were downregulated on both SLActive titanium and titanium–zirconium discs compared to their corresponding SLA surfaces. However, it is difficult to ascertain if these levels were statistically significant in this study. Alfarsi et al. (2014) assessed gene fold changes for IL‐1β and TNF‐α on titanium discs. However, these measures were not reported or displayed within the full‐text article.

Hotchkiss et al. (2019) found that the IL‐4 gene expression was significantly upregulated on titanium–zirconium implants with SLActive compared to SLA surfaces. Similarly, Hotchkiss et al. (2017) found that IL‐10 was upregulated on hydrophilic SLA surfaces compared to hydrophobic surfaces for titanium and titanium–zirconium materials. However, it is unclear if this difference were statistically significant. However, Hamlet et al. (2012) found that downregulation of IL‐4 (−2.73) and IL‐10 (−4.48) genes on titanium discs with SLActive treatment when compared to SLA surfaces, although this was found not to be statistically significant.

*At 72 h*: Alfarsi et al. (2014) found that modSLA surfaces on titanium discs significantly downregulated gene expression of IL‐1β (*p* = .036) and TNF‐α (*p* = .024) in comparison to SLA surfaces. Wang et al. (2019) found that TNF‐α was downregulated on hydrophilic SLA surfaces on titanium and titanium–zirconium discs compared to SLA surfaces (*p* < .05). In addition, IL‐6 was reduced on modSLA surfaces on titanium and titanium–zirconium discs. However, this was only statistically significant (*p* < .05) on titanium surfaces. The expression of IL‐10 was significantly increased on modSLA discs in comparison to SLA discs for both titanium and titanium–zirconium materials (*p* < .05) (Wang et al., 2019).

*At 5 days*: Wang et al. (2019) stated that they had assessed inflammation‐related gene expression on Day 5. However, this was not displayed or reported within the full‐text report.

The findings relating to gene expression of proinflammatory and anti‐inflammatory gene expression are outlined in Table 3.

**Table 3:** Summary of study findings reporting on gene expression on hydrophilic SLA surfaces compared to SLA surfaces.

Authors and year of publication	Hamlet et al. (2012)	Alfarsi et al. (2014)	Hotchkiss et al. (2017)	Hotchkiss et al. (2019)	Wang et al. (2019)
Test materials	Grade II commercially pure titanium discs	Grade II commercially pure titanium discs	Unalloyed titanium (grade not specified) and titanium–zirconia discs	Titanium–zirconium implants	Grade IV commercially pure titanium and titanium–zirconium alloy discs
Surface treatments	SLActive and SLA	modSLA and SLA	modSLA and SLA	SLActive and SLA	modSLA and SLA
Measurement timepoints	24 h after plating	24 and 72 h after plating	24 h after plating	24 h after plating	72 h and 5 days after plating
Findings	24 h: SLActive surface was found to significantly (*p* < .05) downregulate IL‐1β (−11.91) and TNF‐α (−1.86) compared to hydrophobic SLA. In addition, a downregulation of IL‐4 (−2.73) and IL‐10 (−4.48) genes were found to be downregulated on the SLActive surface in comparison to SLA; however, this was not statistically significant.	24 h: *p* Values and direction of gene regulation were not available or discussed for IL‐1β and TNF‐α. 72 h: modSLA had a downregulatory effect on IL‐1β (−1.76) (*p* = .036) and TNF‐α (−1.58) (*p* = .024) when compared to SLA.	24 h: IL‐1β, IL‐6, and TNF‐α were downregulated on modSLA surfaces for both titanium and titanium–zirconia discs. However, it is unclear if this reached statistical significance. IL‐10 showed an increased expression on modSLA surfaces when compared to SLA. However, it was unclear if this was statistically significant. These differences appeared to be independent of the material examined.	24 h: IL‐1β, IL‐6, and TNF‐α gene expression was significantly reduced on SLActive surface when compared to SLA. IL‐4 gene expression was significantly increased on SLActive surfaces in comparison to SLA.	72 h: TNF‐α and IL‐6 were significantly downregulated (*p* < .05) on modSLA when compared to SLA for titanium surfaces. On titanium–zirconium surfaces, TNF‐α was significantly (*p* < .05) downregulated on hydrophilic surfaces. IL‐6 was reduced on titanium–zirconium hydrophilic surfaces, although this was not statistically significant. The expression of IL‐10 was significantly (*p* < .05) increased on modSLA discs in comparison to SLA discs for titanium and titanium–zirconium. 5 days: Gene expression data was not displayed or reported.
Outcome summary	Significant downregulation of proinflammatory genes for IL‐1β and TNF‐α on SLActive surfaces when compared to SLA at 24 h.	Significant downregulation of proinflammatory genes for IL‐1β and TNF‐α on modSLA surfaces when compared to SLA at 72 h.	After 24 h, there was a reduced proinflammatory genetic expression of IL‐1β, IL‐6, and TNF‐α on modSLA surfaces, while IL‐10 was upregulated. However, it is unclear if these changes were statistically significant.	There was a significant downregulation of proinflammatory genes and a significant upregulation of anti‐inflammatory genes at 24 h on SLActive surfaces when compared to SLA.	At 72 h, a significant downregulation of proinflammatory TNF‐α was present on modSLA surfaces. IL‐6 was downregulated on modSLA titanium surfaces only. Anti‐inflammatory IL‐10 was significantly upregulated on titanium and titanium–zirconium modSLA surfaces.

Abbreviations: IL, interleukin; modSLA, modified SLA; SLA, sandblasted large grit, acid‐etched; SLActive, hydrophilic sandblasted large grit, acid‐etched; TNF, tumor necrosis factor‐α.

#### 3.2.2 Cytokine production

*At 24 h or less*: Hotchkiss et al. (2018) found macrophages on modSLA titanium discs; IL‐6 was increased compared to hydrophobic SLA at 6, 18, and 24 h. At the 12‐h timepoint, there was no difference for IL‐6 found between SLA and SLActive surfaces. However, it was difficult to ascertain if these findings individually reached statistical significance. Overall for the 24‐h timepoint, IL‐6 had significantly reduced quantities on SLActive surfaces compared to their hydrophobic counterparts (Hotchkiss et al., 2016, 2017, 2018). Hotchkiss et al. (2017) found that this reduction of IL‐6 was independent of whether titanium or titanium–zirconium was used.

Hotchkiss et al. (2018) found that IL‐1β and TNF‐α were increased on hydrophilic SLA surfaces at 12 h. This pattern switched after 18‐ and 24‐h timepoints showing a reduced production of IL‐1β and TNF‐α on SLActive surfaces compared to hydrophobic SLA. For the 24‐h period, IL‐1β and TNF‐α protein levels were significantly reduced on hydrophilic SLA surfaces compared to hydrophobic SLA surfaces (Hotchkiss et al., 2016, 2017). This finding appeared to be independent of whether titanium or titanium–zirconium was used as the test material (Hotchkiss et al., 2017). Hotchkiss et al. (2018) found that there was a general pattern of reduction of IL ‐1β and TNF‐α, although, for the 24‐h period overall, this finding was not statistically significant.

IL‐4 and IL‐10 levels on SLActive surfaces were increased at 6, 12, 18, and 24 h compared to hydrophobic SLA (Hotchkiss et al., 2018). Hotchkiss et al. (2016, 2017, 2018) found that overall, for the first 24 h, IL‐4 and IL‐10 levels were also significantly increased on SLActive surfaces compared to hydrophobic SLA. Interestingly, the significant increase in IL‐4 and IL‐10 levels appeared to be independent of whether titanium or titanium–zirconium material was used (Hotchkiss et al., 2017).

*At 48 h*: Abaricia et al. (2021) found that IL‐1β, IL‐6, and TNF‐α quantities were significantly reduced on SLActive surfaces compared to SLA for titanium and titanium–zirconium implants. Hotchkiss et al. (2019) demonstrated that SLActive surface treatment produced less IL‐1β, IL‐6, IL‐12, and TNF‐α than SLA surfaces on titanium–zirconium implants. However, these findings were not statistically significant.

Hotchkiss et al. (2019) observed that IL‐4 and IL‐10 quantities were significantly increased on SLActive surfaces than on SLA surfaces for titanium–zirconium implants. This finding is similar to Abaricia et al. (2021), which found significantly increased IL‐4 and IL‐10 levels on SLActive surfaces compared to SLA for both titanium and titanium–zirconium implants.

*At 72 h*: Hotchkiss et al. (2016) found that IL‐1β, IL‐6, and TNF‐α quantities were significantly reduced on modSLA titanium discs compared to hydrophobic SLA. In addition, Hotchkiss et al. (2017) found significantly reduced IL‐6 and TNF‐α for SLActive surfaces on titanium and titanium–zirconium surfaces compared to their corresponding hydrophobic counterparts. However, IL‐1β was only significantly reduced on titanium modSLA compared to SLA and not for titanium–zirconium surfaces. Alfarsi et al. (2014) found IL‐1β and TNF‐α were reduced on modSLA compared to SLA grade II titanium discs. However, whether this difference was statistically significant is unclear.

IL‐4 and IL‐10 quantities were significantly increased on modSLA titanium disc surfaces compared to hydrophobic SLA (Hotchkiss et al., 2016). In addition, these cytokines were also significantly increased on modSLA titanium and titanium–zirconium disc surfaces compared to SLA surface treatments (Hotchkiss et al., 2017).

The findings relating to proinflammatory and anti‐inflammatory protein production are outlined in Table 4.

**Table 4:** Summary of study findings reporting on cytokine production on hydrophilic SLA surfaces compared to SLA surface.

Authors and year of publication	Alfarsi et al. (2014)	Hotchkiss et al. (2016)	Hotchkiss et al. (2017)	Hotchkiss et al. (2018)	Hotchkiss et al. (2019)	Abaricia et al. (2021)
Test materials	Grade II commercially pure titanium discs	Grade II unalloyed titanium discs	Unalloyed titanium (grade not specified) and titanium‐zirconia discs	Grade II unalloyed titanium discs	Titanium‐zirconium implants	Grade IV titanium and titanium–zirconium implants
Surface treatment	modSLA and SLA	modSLA and SLA	modSLA and SLA	SLActive and SLA	SLActive and SLA	SLActive and SLA
Measurement timepoints	72 h after plating	24 and 72 h after seeding	24 and 72 h after plating	6, 12, 18, and 24 h after plating	48 h after plating	48 h after plating
Findings summary	7*2 h*: IL‐1β (−1.76) and TNF‐α (−1.81) were reduced on modSLA when compared SLA. However, it is unclear if this met the level of statistical significance.	*24 h*: IL‐1β, IL‐6, and TNF‐α secretion were significantly (*p* < .05) reduced on modSLA surfaces compared to SLA surfaces. Anti‐inflammatory cytokines IL‐4 and IL‐10 were upregulated on modSLA surfaces in comparison to SLA surfaces (*p* < .05). *72 h*: IL‐1β, IL‐6, and TNF‐α were significantly (*p* < .05) reduced on the modSLA surface compared to the SLA surface. IL‐4 and IL‐10 levels were increased on the modSLA surface when compared to SLA surface (*p* < .05).	*24 h*: IL‐1β, TNF‐α, and IL‐6 were significantly (*p* < .05) reduced on modSLA surfaces when compared to SLA on titanium and titanium–zirconia. IL‐4 and IL‐10 were found at significantly (*p* < .05) increased levels for modSLA compared to SLA on titanium and titanium‐zirconia. *72 h*: IL‐1β, IL‐6, and TNF‐α on titanium modSLA surfaces were significantly (*p* < .05) reduced compared to SLA. IL‐1β on titanium–zirconia did not reach the level of statistical significance. IL‐4 and IL‐10 were significantly increased for modSLA compared to SLA on titanium and titanium–zirconium materials.	There was an overall trend for lower levels of proinflammatory cytokines IL‐1β, IL‐6, and TNF‐α on the SLActive surface compared to the SLA along the measured timepoints. In addition, anti‐inflammatory markers IL‐4 and IL‐10 were also seen to be reduced. 2*4 h*: For the overall time period, there was no statistically significant difference found for IL‐1β, IL‐6, and TNF‐α produced by macrophages on SLActive compared to SLA surfaces. In addition, IL‐4 and IL‐10 were significantly increased on SLActive surfaces compared to SLA surfaces (*p* < .05).	*48 h*: TNF‐α, IL‐1β, IL‐6, and IL‐12 levels were lower on titanium‐zirconium with SLActive when compared to SLA. However, this did not reach statistical significance. Anti‐inflammatory cytokines IL‐4 and IL‐10 were found to be released in significantly (*p* < .05) increased amounts for SLActive compared to SLA.	*48 h*: SLActive titanium and titanium–zirconium surfaces had significantly reduced IL‐1β, IL‐6, and TNF‐α when compared to SLA (*p* < .05). Titanium and titanium–zirconium SLActive surfaces had significantly (*p* < .05) increased anti‐inflammatory proteins IL‐4 and IL‐10 levels present in comparison to SLA.
Outcome summary	There were reduced levels of proinflammatory IL‐1β and TNF‐α were found on modSLA compared to SLA surfaces after 72 h.	Reduced levels of proinflammatory cytokines and increased levels of anti‐inflammatory cytokines on modSLA surfaces were found after 24 and 72 h compared to SLA surfaces.	There were reduced levels of proinflammatory and increased levels of anti‐inflammatory cytokines found on modSLA surfaces compared to SLA surfaces after 24 and 72 h.	There was a reduced quantity of proinflammatory cytokines produced on SLActive surfaces, although this did not reach statistical significance. Anti‐inflammatory cytokines were significantly reduced on SLActive surfaces compared to SLA overall after 24 h.	Significantly increased quantities of anti‐inflammatory cytokines were found on SLActive surfaces compared to SLA surfaces after 48 h. Proinflammatory cytokines were reduced, although they were not statistically significant.	After 48 h, there is a significantly reduced level of proinflammatory cytokines on SLActive surfaces, while anti‐inflammatory cytokines were significantly reduced. These results were independent of the material used.

Abbreviations: IL, interleukin; modSLA, modified SLA; SLA, sandblasted large grit, acid‐etched; SLActive, hydrophilic sandblasted large grit, acid‐etched; TNF, tumor necrosis factor‐α.

#### 3.2.3 Macrophage markers

No studies that met this systematic review's eligibility criteria evaluated macrophage surface markers as an outcome measure during in vitro testing (e.g., CD163).

### 3.3 Quality assessment

The overall body of evidence is low to moderate, given the factors previously discussed and outlined in Supporting Information: Appendix 5. Given this overall quality of evidence, no studies were excluded from this systematic review.

## 4 DISCUSSION

Macrophages are critical in the orchestration of the inflammatory response through the initiation and maintenance of the reaction and its resolution (Fujiwara & Kobayashi, 2005). The inflammatory state of macrophages is essential in regeneration and osseointegration (Trindade et al., 2018a, 2018b). Hamlet et al. (2019) found that SLActive surfaces elicit a macrophage phenotype associated with reduced inflammation and enhanced pro‐osteogenic signalling in rodents. This reduced inflammation and increase of osteogenic signals of macrophages may be in part responsible for an increased bone‐to‐implant contact found on SLActive surfaces when compared to SLA surfaces (Lang et al., 2011; Schwarz et al., 2007).

This systematic review found that using SLActive surfaces in an in vitro setting reduces macrophage proinflammatory gene expression compared to hydrophobic SLA surfaces at 24‐ and 72‐h timepoints, while anti‐inflammatory genes were upregulated. In addition, an overall decrease of proinflammatory macrophage cytokines was found on SLActive surfaces compared to SLA at 24‐, 48‐, and 72‐h time points. Contrastingly, an overall increase of anti‐inflammatory IL‐4 and IL‐10 cytokines was found on SLActive surfaces compared to its hydrophobic counterpart. Overall, these findings show that SLActive surfaces can modulate the macrophage inflammatory response toward an anti‐inflammatory state compared to hydrophobic SLA surfaces. It is usually assumed that there is a correlation between genetic expression and protein production. However, this is not always the case and thus gene expression and cytokine production were both investigated in this systematic review (Greenbaum et al., 2003).

The overall quality of the evidence was low to moderate. There are currently numerous quality assessment tools in the literature, although none assess the critical aspects of in vitro study designs (Tran et al., 2021). The quality assessment tool used in this systematic review, “Template for assessing the quality of simple lab studies,” has the flexibility to be adjusted to different types of in vitro studies. However, this tool does not have a validated grading system. The included studies in this systematic review used macrophages from human and murine sources. To the author's knowledge, there is currently no evidence in the literature to suggest that macrophage origin has impacted the outcomes of these studies. M1 and M2 macrophage phenotypes produce various proinflammatory or anti‐inflammatory cytokines overall in nature, respectively (Krzyszczyk et al., 2018). Interestingly, macrophages have plasticity, allowing them to change their phenotype based on environmental signals, thus changing their physiology and immune response (Lee et al., 2019; Mosser & Edwards, 2008). However, none of the included studies in this systematic review tested for macrophage phenotypes before seeding onto the test surfaces, which may have impacted the final cytokine gene expression and cytokine production.

The in vitro nature of the included studies in this review allows researchers to investigate in isolation the impact of surface treatments on the macrophage inflammatory response. However, this isolated interaction between macrophages and the test surface does not replicate the sequence of the multicellular in vivo process of osseointegration (Terheyden et al., 2012). In addition, protein adsorption onto the implant surface is one of the first processes to occur in vivo and regulates the downstream sequence of healing events (Lee et al., 2021). However, in the included in vitro studies this complex process is not replicated.

All of the included studies used commercially pure titanium and/or titanium–zirconium as their test materials. In addition, all studies used SLActive surface treatments and a hydrophobic SLA comparator on their respective test materials. However, average surface roughness appeared to be increased for test materials in a disc form compared to implant form (Hotchkiss et al., 2017, 2019). This difference in surface roughness may have an impact on the macrophage inflammatory response (Hotchkiss et al., 2016). However, surface roughness did not vary between materials used within each individual study.

All studies in this systematic review investigating gene expression used reverse transcription‐polymerase chain reaction testing (Alfarsi et al., 2014; Hamlet et al., 2012; Hotchkiss et al., 2017, 2019; Wang et al., 2019). This method is widely used for gene expression analysis due to its high level of accuracy, sensitivity, specificity, and reproducibility (Zhang et al., 2017). Five out of the six studies which quantified the level of cytokines produced used enzyme‐linked immunosorbent assays (ELISA) to determine the levels of cytokines produced (Abaricia et al., 2021; Hotchkiss et al., 2016, 2017, 2018, 2019). ELISA assays are a specific and sensitive tool for studying cytokines during in vitro studies (Chiswick et al., 2012). The proteome profile kit used by Alfarsi et al. (2014) was a membrane‐based antibody array, which is highly sensitive, specific, and a robust testing method for complex proteomes (Wilson et al., 2015). In addition, Alfarsi et al. (2014) used chemiluminescent detection, which enables fold‐change reading (Skalnikova et al., 2017).

Abaricia et al. (2021) found SLActive‐treated titanium and titanium–zirconium implants placed into inguinal fat pads of mice had significantly more M2 macrophage quantities compared to implants with SLA surface treatment after 3 days. M1 macrophages were present in lesser quantities on titanium–zirconium with SLActive than SLA surface treatment. However, no statistical difference in the amounts of M1 macrophages between titanium with SLA and SLActive treatment was found. Similarly, Lee et al. (2017) found that the M1/M2 macrophage ratio was significantly higher on SLA than on the modSLA surface on Days 1 and 4 in healthy rats. In diabetic rats, a higher M1/M2 macrophage ratio on SLA surfaces was present on Day 4 compared to healthy rats. However, no significant differences in the M1/M2 ratio were present between surface treatments in the healthy and diabetic groups on Day 7. In addition, Lee et al. (2021) found that the ratio of M1/M2 macrophages was significantly (*p* < .01) reduced on modSLA surfaces in comparison to SLA on Days 4 and 7 of healing when titanium discs were placed in the calvarium in rats. This study also found that modSLA surfaces promoted an M2 macrophage phenotype while reducing proinflammatory cytokine production.

Hamlet et al. (2019) used SLA and modSLA grade II commercially pure titanium discs seeded with either M1 or M2 polarized rodent macrophages. M1 polarized proinflammatory macrophages produced significantly less IL‐1β on modSLA than SLA surfaces and a marked CD163 protein expression. M2 polarized macrophages had significantly increased amounts of IL‐10 on modSLA compared to SLA surfaces. This study found that M1 macrophages placed on a modSLA surface promoted an M2‐like phenotype and cytokine release, while M2 macrophages on SLA surfaces promoted an M1 macrophage phenotype and cytokine profile. This study demonstrates the ability of macrophages to undergo phenotypic changes depending on the surface treatment on which they are placed, with SLActive surface promoting an M2 macrophage phenotype.

This systematic review shows that SLActive surfaces on commercially pure titanium or titanium–zirconium materials can modulate the initial macrophage inflammatory response toward an anti‐inflammatory reaction in an in vitro setting. Diabetic wound healing has been associated with a dysregulated M1 macrophage phenotype, while normal healing is associated with an M2 macrophage phenotype (Louiselle et al., 2021). However, implant therapy using titanium–zirconium dental implants with a SLActive surface treatment has been shown to be 100% successful in well‐controlled and poorly controlled type two diabetes patients, although these studies have short clinical follow‐up times (Cabrera‐Domínguez et al., 2020; Latimer et al., 2022). Further studies are required to determine the clinical benefit dental implants with SLActive surface treatment may have in patients with diabetes and other medical conditions which may predispose patients toward an M1 macrophage phenotype during healing.

## 5 CONCLUSIONS

Considering the limitations within this systematic review, a differing macrophage inflammatory response to hydrophilic SLA surfaces compared to hydrophobic SLA on titanium or titanium–zirconium surfaces during in vitro studies is present. The genetic expression of macrophage proinflammatory cytokines on SLActive surfaces overall appears reduced for the studied time points compared to SLA surface, while gene expression for anti‐inflammatory cytokines was increased on SLActive surfaces. Similarly, proinflammatory cytokines produced by macrophages on SLActive surfaces are reduced compared to SLA surfaces. In addition, anti‐inflammatory cytokines are increased on SLActive surfaces compared to SLA surfaces. However, the primary investigator (E. D.) stresses that the conclusion drawn from this systematic review can only be applicable in an in vitro environment.

The in vitro study design has inherent limitations in replicating the in vivo healing response (Salvi et al., 2015). Therefore, further in vivo studies are required to investigate the early macrophage inflammatory response towards titanium and titanium–zirconium materials with SLActive and SLA surface treatments. In addition, future developments in dental implant surface modification may allow for the specific biological targets to enhance an M2 macrophage phenotype (Hachim et al., 2017).

The hydrophilic SLA surfaces of implants have a decreased osseointegration healing timeframe which may allow for faster prosthetic loading for patients (Lang et al., 2011). Further investigation of the impact of the macrophage inflammatory state on the functioning of osteoblasts would enhance our understanding of the possible processes behind this and its potential impact on osseointegration healing timeframes. There may be a theoretical benefit in using dental implants with a SLActive surface treatment in patients with medical conditions with a proinflammatory disposition or a macrophage dysfunction, which may be predisposed to early implant failure (Dubey et al., 2013). However, further clinical studies are required in this area before a clinical recommendation can be made.

## AUTHOR CONTRIBUTIONS

*Conception and design*: Eamonn Donohoe and Fadi Barrak. *Acquisition of data*: Eamonn Donohoe. *Analysis and interpretation of data*: Eamonn Donohoe. *Drafting the manuscript*: Eamonn Donohoe and Rawan Kahatab. *Reviewing and editing the manuscript*: Fadi Barrak. All authors gave final approval for all aspects of this work.

## CONFLICT OF INTEREST STATEMENT

The authors declare no conflict of interest.

## ETHICS STATEMENT

Ethical approval was obtained by the Ethics, Integrity and Governance Unit of the University of Central Lancashire.

## Supporting information

Supplementary information.

Supplementary information.

Supplementary information.

Supplementary information.

Supplementary information.

## Data Availability

The data that supports the findings of this study are available in the Supporting Information: Material of this article.
