# Supplementary material for: A systematic review comparing the macrophage inflammatory response to hydrophobic and hydrophilic sandblasted large grit, acid‐etched titanium or titanium–zirconium surfaces during in vitro studies
Source: Clin Exp Dent Res. 2023 Mar 29;9(3):437–48. doi: 10.1002/cre2.730 (PMC10280619; doi:10.1002/cre2.730)
Supplement: Supplementary file 1 — Supplementary information. [file CRE2-9-437-s002.docx]

Appendix 1: Template for assessing the quality of simple lab studies

Materials

Is the quality of the test material clearly stated?  Yes No

Is the quality of the test material sufficient? Yes No Unclear

Is it clear how the test material was manufactured?  Yes No

Is the makeup of the comparator material clearly stated?  Yes No Unclear

Is the makeup of the comparator material appropriate? Yes No

Was surface hydrophilicity evaluated?   Yes No

Was surface roughness evaluated?   Yes No

Is the source of macrophage clearly stated?  Yes No

Design

Was macrophage differentiation performed appropriately? Yes No Unclear

Is it clear which factors are being studied? Yes No

Was there both an experimental and comparator group?  Yes No Unclear

Was outcome assessment appropriate?   Yes No

Were technical replicates performed? Yes No Unclear

Were experimental replicates performed? Yes No Unclear

Was measurement consistent across experimental groups?  Yes No Unclear

Was measurement consistent for repeated experiments?  Yes No Unclear

Were test methods well-established?  Yes No Unclear

Was the outcome measured directly?  Yes No Unclear

Was the outcome measured objectively?  Yes No Unclear

Is it clear who undertook key parts of the experiment?  Yes No

Were assessors blinded as to the surface treatment?  Yes No Unclear

Analysis/Results

Was sample loss at key points described?  Yes No

Were appropriate statistical tests used? (parametric/non-parametric)  Yes No Unclear

Does data presented align with analyses conducted   Yes No Unclear

Is variability within each condition reported?  Yes No Unclear

Source of funding

May the source of funding have invested interests in the outcomes?  Yes No Unclear

Were the materials supplied by parties with invested interests in the outcomes? Yes No Unclear

Relevance

Are the test conditions clinically relevant?   Yes No

This tool is based on the following sources, as well as expert discussion:

[Krithikadatta](https://www.ncbi.nlm.nih.gov/pubmed/?term=Krithikadatta%20J%5BAuthor%5D&cauthor=true&cauthor_uid=25125839) J, [Gopikrishna](https://www.ncbi.nlm.nih.gov/pubmed/?term=Gopikrishna%20V%5BAuthor%5D&cauthor=true&cauthor_uid=25125839) V, & [Datta](https://www.ncbi.nlm.nih.gov/pubmed/?term=Datta%20M%5BAuthor%5D&cauthor=true&cauthor_uid=25125839) M (2014). CRIS Guidelines (Checklist for Reporting *In-vitro* Studies): A concept note on the need for standardized guidelines for improving quality and transparency in reporting *in-vitro* studies in experimental dental research. *Journal of Conservative Dentistry.*17(4), pp. 301–304.  
 

OHAT Risk of Bias Rating Tool for Human and Animal Studies (2015) <https://ntp.niehs.nih.gov/ntp/ohat/pubs/riskofbiastool_508.pdf>
