# Supplementary material for: A systematic review comparing the macrophage inflammatory response to hydrophobic and hydrophilic sandblasted large grit, acid‐etched titanium or titanium–zirconium surfaces during in vitro studies
Source: Clin Exp Dent Res. 2023 Mar 29;9(3):437–48. doi: 10.1002/cre2.730 (PMC10280619; doi:10.1002/cre2.730)
Supplement: Supplementary file 2 — Supplementary information. [file CRE2-9-437-s004.docx]

| # | Keyword/MeSH | MEDLINE | DOSS | WoS |
| --- | --- | --- | --- | --- |
|  | Search Date | 02/02/2022 | 02/02/2022 | 02/02/2022 |
| 1 | Macrophage | 325,283 | 2,004 | 353,141 |
| 2 | Phagocyte | 30,330 | 129 | 21,579 |
| 3 | M1 | 45,052 | 378 | 67,531 |
| 4 | M2 | 41,391 | 366 | 73,213 |
| 5 | MH macrophages | 140,468 | 785 | 353,611 |
| 6 | #1 OR #2 OR #3 OR #4 OR #5 | 404,915 | 2,506 | 470,780 |
|  |  |  |  |  |
| 7 | Surface modification | 21,734 | 716 | 187,219 |
| 8 | Surface chemistry | 20,520 | 1,069 | 1,078,356 |
| 9 | Surface energy | 28,018 | 417 | 841,140 |
| 10 | Wettability | 12,286 | 375 | 53,094 |
| 11 | Hydrophil* | 104,980 | 758 | 148,551 |
| 12 | Surface characteristics | 14,894 | 928 | 418,098 |
| 13 | Surface characteri?ation | 6,965 | 345 | 266,426 |
| 14 | SLA | 4,815 | 606 | 14,989 |
| 15 | Sandblasted large grit acid-etched | 159 | 106 | 197 |
| 16 | Sandblasted and acid-etched | 601 | 417 | 911 |
| 17 | SLActive | 178 | 177 | 199 |
| 18 | modSLA | 96 | 49 | 90 |
| 19 | Surface properties | 149,920 | 1,772 | 907,880 |
| 20 | MH wettability | 4,567 | 108 | 53,094 |
| 21 | MH surface properties | 122,259 | 968 | 907,880 |
| 22 | #7 OR #8 OR #9 OR #10 OR #11 OR #12 OR #13 OR #14 OR #15 OR #16 OR #17 OR #18 OR #19 OR #20 OR #21 | 316,430 | 5,102 | 2,543,762 |
|  |  |  |  |  |
| 23 | Titanium | 69,467 | 10,514 | 267,324 |
| 24 | Titanium alloy | 6,908 | 1,648 | 68,204 |
| 25 | Titanium-zircon* | 234 | 132 | 1,068 |
| 26 | Ti | 81,873 | 4,857 | 319,519 |
| 27 | TiZ* | 5,692 | 428 | 29,587 |
| 28 | Roxolid | 45 | 49 | 41 |
| 29 | MH Titanium | 42,898 | 5,937 | 267,324 |
| 30 | #23 OR #24 OR #25 OR #26 OR #27 OR #28 OR #29 | 124,857 | 11,586 | 536,881 |
|  |  |  |  |  |
| 31 | Inflam* | 1,242,122 | 30,733 | 1,303,925 |
| 32 | Response | 3,282,744 | 25,645 | 4,533,074 |
| 33 | Activat* | 1,944,210 | 9,453 | 2,690,789 |
| 34 | Immunomodulat* | 75,510 | 505 | 78,123 |
| 35 | Cytokine | 452,298 | 4,392 | 291,797 |
| 36 | Polari* | 196,543 | 1,284 | 784,018 |
| 37 | Modulat* | 792,889 | 3,520 | 1,292,312 |
| 38 | Gene expression | 1,302,128 | 5,353 | 1,687,818 |
| 39 | Protein | 5,057,177 | 17,023 | 4,225,860 |
| 40 | Behaviour | 326,655 | 15,580 | 4,298,249 |
| 41 | Inflammatory mediators | 50,247 | 873 | 68,153 |
| 42 | Genetic expression | 57,303 | 2,948 | 249,370 |
| 43 | IL-12 | 20,166 | 134 | 28,308 |
| 44 | Interleukin 12 | 8,295 | 39 | 51,127 |
| 45 | IL-1b | 1,636 | 114 | 2,418 |
| 46 | IL-1β | 49,842 | 1,237 | 0 |
| 47 | Interleukin 1 beta | 11,041 | 36 | 87,389 |
| 48 | IL-6 | 130,054 | 1,600 | 135,743 |
| 49 | Interleukin 6 | 45,841 | 632 | 149,459 |
| 50 | TNF-a | 2,277 | 2,163 | 7,594 |
| 51 | TNF-α | 84,744 | 1,416 | 0 |
| 52 | tumor necrosis factor alpha | 157,719 | 1,253 | 152,399 |
| 53 | Tumour Necrosis Factor alpha | 59,329 | 1,253 | 152,253 |
| 54 | IL-4 | 44,718 | 294 | 48,691 |
| 55 | Interleukin 4 | 14,322 | 114 | 96,856 |
| 56 | IL-10 | 62,486 | 573 | 67,879 |
| 57 | Interleukin 10 | 17,460 | 121 | 93,271 |
| 58 | CD163 | 4,314 | 65 | 4,743 |
| 59 | Surface marker | 34,745 | 237 | 59,368 |
| 60 | Markers | 877,635 | 7,759 | 1,034,536 |
| 61 | MH Gene expression | 205,238 | 3,202 | 1,687,818 |
| 62 | MH Macrophage activation | 15,148 | 25 | 100,545 |
| 63 | MH Macrophage Inflammatory Proteins | 2,465 | 14 | 44,984 |
| 64 | MH Inflammation | 183,136 | 5,712 | 695,358 |
| 65 | MH Inflammation Mediators | 37,357 | 16 | 55,272 |
| 66 | MH Cytokines | 164,765 | 2,215 | 299,356 |
| 67 | #31 OR #32 OR #33 OR #34 OR #35 OR #36 OR #37 OR #38 OR #39 OR #40 OR #41 OR #42 OR #43 OR #44 OR #45 OR #46 OR #47 OR #48 OR #49 OR #50 OR #51 OR #52 OR #53 OR #54 OR #55 OR #56 OR #57 OR #58 OR #59 OR #60 OR #61 OR #62 OR #63 OR #64 OR #65 OR #66 | 9,914,729 | 87,032 | 15,859,773 |
|  |  |  |  |  |
| 68 | **#6 AND #22 AND #30 AND #67** | 255 | 19 | 632 |

**Appendix 2: Search Strategy Results**
