# Supplementary material for: A systematic review comparing the macrophage inflammatory response to hydrophobic and hydrophilic sandblasted large grit, acid‐etched titanium or titanium–zirconium surfaces during in vitro studies
Source: Clin Exp Dent Res. 2023 Mar 29;9(3):437–48. doi: 10.1002/cre2.730 (PMC10280619; doi:10.1002/cre2.730)
Supplement: Supplementary file 3 — Supplementary information. [file CRE2-9-437-s003.docx]

Appendix 3: Table of excluded studies

| **Authors and year of publication** | **Title of study** | **Type of publication** | **Reason for exclusion** |
| --- | --- | --- | --- |
| Stanford (2010) | Surface modification of biomedical and dental implants and the processes of inflammation, wound healing and bone formation | Review article | Review article |
| Alfarsi *et al.* (2015) | The Effect of Platelet Proteins Released in Response to Titanium Implant Surfaces on Macrophage Pro-Inflammatory Cytokine Gene Expression | *In-vitro* study | Inappropriate study design:  Platelet proteins were present on titanium surfaces prior to macrophage seeding |
| Lee *et al.* (2017) | The influence of titanium surface characteristics on macrophage phenotype polarization during osseous healing in type I diabetic rats: a pilot study | *In-vivo* study | Inappropriate study design:  *in-vivo* study design |
| Lv *et al.* (2018) | Unveiling the Mechanism of Surface Hydrophilicity-Modulated Macrophage Polarization | *In-vitro* study | Unsuitable surface treatments |
| Wang *et al.* (2018) | The role of macrophage polarization on fibroblast behavior-an in vitro investigation on titanium surfaces | *In-vitro* study | Unsuitable surface treatments |
| Hamlet *et al.* (2019) | Hydrophilic titanium surface-induced macrophage modulation promotes pro-osteogenic signaling | *In-vitro* study | Polarization of macrophage prior to seeding:  Polarization of macrophages towards a M1 or M2 phenotype took place prior to seeding on surface substrates |
| Lee *et al.* (2021) | Re-establishment of macrophage homeostasis by titanium surface modification in type II diabetes promotes osseous healing | *In-vivo* study | Inappropriate study design:  *in-vivo* study design |
