# Supplementary material for: A systematic review comparing the macrophage inflammatory response to hydrophobic and hydrophilic sandblasted large grit, acid‐etched titanium or titanium–zirconium surfaces during in vitro studies
Source: Clin Exp Dent Res. 2023 Mar 29;9(3):437–48. doi: 10.1002/cre2.730 (PMC10280619; doi:10.1002/cre2.730)
Supplement: Supplementary file 4 — Supplementary information. [file CRE2-9-437-s001.docx]

| **Authors and year of publication** | **Hamlet *et al* (2012)** | **Alfarsi *et al* (2014)** | **Hotchkiss *et al* (2016)** | **Hotchkiss *et al* (2017)** | **Hotchkiss *et al* (2018)** | **Hotchkiss *et al* (2019)** | **Wang *et al* (2019)** | **Abaricia *et al* (2021b)** |
| --- | --- | --- | --- | --- | --- | --- | --- | --- |
| **Title of study** | The effect of hydrophilic titanium surface modification on macrophage inflammatory cytokine gene expression | Titanium surface hydrophilicity modulates the human macrophage inflammatory cytokine response | Titanium surface characteristics, including topography and wettability, alter macrophage activation | Dental implant surface chemistry and energy alter macrophage activation in vitro | Macrophage response to hydrophilic biomaterials regulates MSC recruitment and T-helper cell populations | Novel in vitro comparative model of osteogenic and inflammatory cell response to dental implants | Macrophage behavior and interplay with gingival fibroblasts cultured on six commercially available titanium, zirconium, and titanium-zirconium dental implants | Surface characteristics on commercial dental implants differentially activate macrophages in vitro and in vivo |
| **Journal of study** | Clinical Oral Implants Research | Journal of Biomedical Materials Research Part A | Acta Biomaterialia | Clinical Oral Implants Research | Biomaterials | Dental Materials | Clinical Oral Investigations | Clinical Oral Implants Research |
| **Publication type** | Full-text paper | Full-text paper | Full-text paper | Full-text paper | Full-text paper | Full-text paper | Full-text paper | Full-text paper |
| **Aims** | To determine the in vitro effect of hydrophilic surface modification on the expression of pro-inflammatory cytokines from adherent macrophages. | To investigate the modulatory effect of a hydrophilic titanium surface on the inflammatory cytokine expression profile in a human macrophage cell line (THP-1). | To examine the effect of surface modifications on macrophage activation, polarization and cytokine production. | To determine the effects of dental implant surface chemistry and energy on macrophage activation in vitro. | To examine whether implant surface properties influence initial events following placement including T-cell activation and MSC recruitment and whether macrophages are instrumental in the phenomena. | To characterize the macrophage inflammatory response and MSC osteogenesis across different commercially available implants in vitro. | To investigate how macrophages behave on various dental implant surfaces and thereafter to investigate their effect on soft tissue cells | To examine macrophage response to commercially available Ti or Ti alloy implants with comparable roughness and varying hydrophilicity. |
| **Location of study** | Australia | Australia | United States of America | United States of America | United States of America | United States of America | China | United States of America |
| **Study design** | In-vitro | In-vitro | In-vitro | In-vitro | In-vitro and in-vivo components | In-vitro | In-vitro | In-vitro and in-vivo components |
| **Test materials** | Grade II commercially pure titanium discs | Grade II commercially pure titanium discs | Grade II unalloyed titanium discs | Unalloyed titanium (grade not specified) and titanium-zirconia discs | Grade II unalloyed titanium discs | Titanium-zirconium implants | Grade IV commercially pure titanium and titanium-zirconium alloy discs | Grade IV Titanium and titanium-zirconium implants |
| **Control/comparator material** | Glass cover slip | Polished titanium | TCPS | TCPS | TCPS | TCPS – Gene expression  SLA – Cytokine production | Smooth-pickled titanium | SLA |
| **Size of material** | 1mm thick, 15mm diameter | 1 mm thick, 15 mm diameter | 1mm thick, 15mm diameter | 1mm thick, 15mm diameter | 1mm thick and 15mm diameter discs | Whole implant - implant dimensions not available | 1mm thick and 15mm diameter discs | Whole implant  Titanium: 4.1x12mm  Ti-Zr: 2.9x10mm |
| **Surface treatments** | SLActive and SLA | modSLA and SLA | modSLA and SLA | modSLA and SLA | SLActive and SLA | SLActive and SLA | modSLA and SLA | SLActive and SLA |

Appendix 4: Study characteristics and findings

| **(…continued)** | **Hamlet *et al* (2012)** | **Alfarsi *et al* (2014)** | **Hotchkiss *et al* (2016)** | **Hotchkiss *et al* (2017)** | **Hotchkiss *et al* (2018)** | **Hotchkiss *et al* (2019)** | **Wang *et al* (2019)** | **Abaricia *et al* (2021b)** |
| --- | --- | --- | --- | --- | --- | --- | --- | --- |
| **Source of materials** | Surfaces were provided by Institut Straumann AG, Basel, Switzerland. | Surfaces were provided by Institut Straumann AG, Basel, Switzerland. | Surfaces were provided by Institut Straumann AG, Basel, Switzerland. | Surfaces were provided by Institut Straumann AG, Basel, Switzerland. | Surfaces were provided by Institut Straumann AG, Basel, Switzerland. | Institut Straumann AG, Basel, Switzerland provided the implants in this study. | All discs were provided by Institut Straumann AG, Basel, Switzerland | Implants were supplied by the Institut Straumann AG, Basel, Switzerland. |
| **Macrophage origin** | RAW 264.7 cells, a murine leukaemic monocyte cell line | Human acute monocytic leukemia cell-line THP-1 | Isolated from femurs of 6–8-week-old male C57BL/6 mice | Isolated from the femoral  bone marrow of 6- to 8-week-old male C57Bl/6 mice | Isolated from femurs of 8–12-week-old male C57BL/6 mice | Isolated from bone-marrow of 10–12-week-old male C57Bl/6 mice | Murine-derived macrophage cell line RAW 264.7 | Human macrophages were derived from human peripheral blood monocytes |
| **Culture methods**  **prior to cell plating** | - | The monocytes were allowed to proliferate in RPMI-1460 supplemented with 10% fetal bovine serum  and 1% penicillin/ streptomycin at 37C in a 5% CO2 atmosphere.  Monocytes were induced  to differentiate into macrophages by incubation with 100ng/mL phorbol myristate acetate for 2 days followed by a further 2 days  incubation without PMA.  Adherent macrophages were then detached with 0.25% trypsin/EDTA. | Bone marrow cells were flushed from the femurs using Dulbecco’s phosphate-buffered saline. Red blood cells were lysed from the bone marrow extract with ACK Lysing Buffer. Cells were counted and plated in a 75 cm2 flask at a density of 500,000 cells/mL in 10 mL RPMI 1640 media supplemented with 10% fetal bovine serum, 50 U/mL penicillin-50 lg/mL streptomycin, and 30 ng/mL macrophage M-CSF. Cells were cultured at 37C, 5% CO2, and 100% humidity. Fresh media supplemented with M-CSF was added after four days.  After a total of seven days the macrophages were seeded. | Marrow was flushed from the canal using Dulbecco’s phosphate-buffered saline and red blood cells lysed from the bone marrow  extract with ACK Lysis Buffer. Cells were cultured in a 75 cm2  flask at a density of 500,000 cells/ml in RPMI 1640 supplemented with 10% fetal bovine serum, 50 U/ml  penicillin–streptomycin, and 30 ng/ml of macrophage colony-stimulating factor at 37°C and 5% CO2 for 7 days to achieve a homogenous naïve macrophage population. The medium was exchanged after 4 days of culture.  Naïve macrophages were sub-passaged and seeded onto the disc surfaces. | Bone marrow cells were flushed from the femurs  using Dulbecco's phosphate-buffered saline. Red blood cells were removed from flushed bone  marrow by addition of ACK Lysing Buffer. Cells were counted and plated in a 175 cm2 flask at a density of 500,000 cells/mL in 30 mL RPMI 1640 media supplemented with 10% fetal bovine serum, 50U/mL penicillin-50μg/mL streptomycin, and 30ng/mL macrophage colony-stimulating factor. Cells were cultured at 37 °C, 5% CO2, and 100% humidity. Fresh media supplemented with M-CSF was added after four days.  After seven days of exposure to growth factors,  macrophages were passaged with Accutase at room temperature for 1 h. | Bones were removed, flushed with PBS, and plated at a density of 500,000 cells/mL after red cell lysis. Naïve macrophages were generated in culture in RPMI 1640 supplemented with 10% fetal bovine serum 50U/mL penicillin- 50ug/mL streptomycin, 50U/mL sodium pyruvate, and 30ng/mL macrophage colony stimulating factor.  After seven days, macrophages were sub-passaged using non-enzymatic methods for experiments. | - | Human macrophages were derived from purchased human peripheral blood monocytes and cultured in RPMI 1,640 supplemented with 10% fetal bovine serum, 50U/mL penicillin-50μg/ml streptomycin, and 50 ng/ml macrophage colony-stimulating factor to generate macrophages. Fresh media supplemented with M-CSF  was added after four days.  After seven days of exposure to M-CSF, macrophages were passaged with Accutase for experiments. |

| **(…continued)** | **Hamlet *et al* (2012)** | **Alfarsi *et al* (2014)** | **Hotchkiss *et al* (2016)** | **Hotchkiss *et al* (2017)** | **Hotchkiss *et al* (2018)** | **Hotchkiss *et al* (2019)** | **Wang *et al* (2019)** | **Abaricia *et al* (2021b)** |
| --- | --- | --- | --- | --- | --- | --- | --- | --- |
| **Cell plating** | Cells were seeded in triplicate onto the discs in 24-well tissue culture plates at a density of 5x10^4^ cells/well and cultured in RPMI 1640 medium supplemented with 1% penicillin/streptomycin  and 10% heat inactivated iron supplemented calf serum at 37°C in a humidified 5% CO2 atmosphere. | Cells were seeded onto titanium discs at a density of 1 x 105 cells per discs in a 24-well tissue culture plates in triplicate. | Macrophages were seeded onto discs in 24 tissue culture well plates at a density of 20,000 cells per cm2 | Naïve macrophages were sub-passaged and seeded onto the disc surfaces at a density of 50,000 cells/cm^2^ | Macrophages were seeded onto Ti surfaces at a density of 50,000 cells/ cm^2^ for each experiment on discs in a 24-well-plate with six independent cultures per surface. | Macrophages were seeded directly onto the implant surface at a density of 200,000 cells/cm^2^ | Cells were seeded on surfaces in 24-well culture plates containing Dulbecco’s modified Eagle’s medium supplemented with 10% fetal bovine serum and 1% penicillin/streptomycin at 37 °C in a humidified 5% CO2 atmosphere at a density of 10000 cells per well. | Human macrophages were plated directly onto the implant surface at a density of 200,000 cells/cm2 |
| **Outcome measures and timepoints:** | Gene expression: Measurement at 24 hours after seeding | Gene expression: Measurement at 24 hours and 72 hours after seeding  Cytokine production: Day 3 after seeding | Cytokine production: Measurement 24 and 72 hours after seeding | Gene expression: measurement after 24 hours after seeding  Cytokine production: after Day 1 and Day 3 of seeding | Cytokine production: After 6, 12, 18 and 24 hours of seeding. The overall 24-hour total was quantified. | Gene expression: After 24 hours of seeding.  Cytokine production: After 24 hours and 48 hours of seeding | Gene expression: Three and five days after seeding  Cytokine production: 48 hours after seeding | Cytokine production: 48 hours after seeding |
| **Measurement of outcome:** | Gene expression:  Murine PCR Array (PAMM-011), (SABiosciences). | Gene expression:  RT2 ProfilerTM PCR Array System (SABiosciences, VIC, Australia).  Cytokine production:  A Proteome Profiler Kit (R&D Systems, MN). | Cytokine production:  ELISA (PeproTech). | Gene expression:  StepOnePlus Real-  Time PCR System (Life Technologies)  Cytokine production:  ELISA (PeproTech). | Cytokine production:  ELISA. | Gene expression:  Custom Bio-Rad PCR arrays (Bio-Rad, Hercules, CA) were designed.  Cytokine production:  ELISA. | Gene expression:  Real-time PCR. | Cytokine production:  ELISA (BioLegend). |
| **Statistical analysis software:** | Not stated | Not stated | JMP pro11 software | JMP pro11 software | Prism GraphPad 5.0 and JMP Pro software. | Prism GraphPad V7 software | GraphPad Software v.6 | Prism GraphPad V7 software |
| **Replicates and information on number of test surfaces:** | Three replicate experiments were performed each with six individual cell cultures per surface. | Experiments were not repeated. | Experiments were performed at least twice to ensure consistent results.  Each cell study experiment was conducted with six independent cultures per surface. | Experiments were repeated three times to validate results.  Each experiment was conducted with six independent culture per surface. | In vitro experiments were conducted with six independent cultures per surface.  However, there was no statement present whether the experiments were repeated | Experiments were performed three times.  The experiments were conducted with six implants being used for each implant type. | The experiments were performed in triplicate with three independent experiments. | All experiments were repeated at least twice to confirm results.  There was a sample size of six for each implant type. |

| **(…continued)** | **Hamlet *et al* (2012)** | **Alfarsi *et al* (2014)** | **Hotchkiss *et al* (2016)** | **Hotchkiss *et al* (2017)** | **Hotchkiss *et al* (2018)** | **Hotchkiss *et al* (2019)** | **Wang *et al* (2019)** | **Abaricia *et al* (2021b)** |
| --- | --- | --- | --- | --- | --- | --- | --- | --- |
| **Statistical methods:** | No assessment of data normality was conducted  Significance was assessed by ANOVA. All pair-wise comparisons were performed by the post hoc tests of Tukey. | No assessment of data normality was conducted  Gene expression data was analysed using the student’s t-test. Fold-change differences in gene expression for each pair-wise comparison between the different titanium surfaces were also calculated. | No assessment of data normality was conducted.  A one-factor ANOVA was used and multiple comparisons between the group means were made using the Tukey-HSD method. | Data were determined to be reasonably normally distributed based on assessment of QQ plots.  A one-factor ANOVA was used. Multiple comparisons between group means were then made using the Tukey HSD method | Normality of in vitro data was determined by assessment of QQ plots.  A one-factor ANOVA was used. Multiple comparisons between the group means were made using the Tukey-HSD method. | Data was found to be normally distributed based on analysis of QQ plots for each group.  A one-factor ANOVA was used, and then multiple comparisons were made between group means with TUKEY-HSD. | No assessment of data normality was conducted.  Statistical analysis was performed by one- and two-way ANOVA with a Bonferroni test. | Data were first subjected to Shapiro–Wilk normality test. The results showed that the data was normally distributed.  A one-factor ANOVA was used with post-hoc  TUKEY-HSD for multiple comparisons. |
| **Level of statistical significance:** | p-value < 0.05 | p-value < 0.05 | p-value < 0.05 | p-value < 0.05 | p-value < 0.05 | p-value < 0.05 | p-value < 0.05 | p-value < 0.05 |
| **Results**  **Gene expression:** | **24 hours:** SLActive surface was found to significantly (p < 0.05) down-regulate IL1b (-11.91) and TNF-α (-1.86) compared to hydrophobic SLA.  In addition, a downregulation of IL-4 (-2.73) and IL-10 (-4.48) genes were found to be downregulated on the SLActive surface in comparison to SLA, however this were not statistically significant. | **24 hours:**  p-values and direction of gene regulation were not available or discussed for IL-1β and TNF-α.  **72 hours:** modSLA had a down-regulatory effect on IL-1β (-1.76) (p = 0.036) and TNF-α (-1.58) (p = 0.024) when compared to SLA. | Not assessed | **24 hours:** IL-1β, IL-6 and TNF-α were downregulated on modSLA surfaces for both titanium and titanium-zirconia discs. However, it is unclear if this reached statistical significance.  IL-10 showed an increased expression on modSLA surfaces when compared to SLA. However, it was unclear if this was statistically significant.  These differences appeared to be independent of material examined. | Not assessed | **24 hours:** IL-1β, IL-6 and TNF-α gene expression was significantly reduced on SLActive surface when compared to SLA.  IL-4 gene expression was significantly increased on SLActive surfaces in comparison to SLA. | **72 hours:** TNF-α and IL-6 were significantly downregulated (p<0.05) on modSLA when compared to SLA for titanium surfaces. On titanium-zirconium surfaces, TNF-α was significantly (p<0.05) downregulated on hydrophilic surfaces. IL-6 was reduced on titanium-zirconium hydrophilic surfaces, although this was not statistically significant.  The expression of IL-10 was significantly (p<0.05) increased on modSLA discs in comparison to SLA discs for titanium and titanium-zirconium.  **Five days:** Gene expression data was not displayed or reported. | Not assessed |

| **(…continued)** | **Hamlet *et al* (2012)** | **Alfarsi *et al* (2014)** | **Hotchkiss *et al* (2016)** | **Hotchkiss *et al* (2017)** | **Hotchkiss *et al* (2018)** | **Hotchkiss *et al* (2019)** | **Wang *et al* (2019)** | **Abaricia *et al* (2021b)** |
| --- | --- | --- | --- | --- | --- | --- | --- | --- |
| **Results**  **Cytokine production:** | Not assessed | **72 hours:** IL-1β (-1.76) and TNF-α (-1.81) were reduced on modSLA when compared SLA. However, it is unclear if this met the level of statistical significance. | **24-hours:** IL-1β, IL-6, and TNF-α secretion were significantly (p<0.05) reduced on modSLA surfaces compared to SLA surfaces. Anti-inflammatory cytokines IL-4 and IL-10 were up-regulated on modSLA surfaces in comparison to SLA surfaces (p<0.05).  **72-hours:** IL-1β, IL-6, and TNF-α were significantly (p<0.05) reduced on the modSLA surface compared to the SLA surface. IL-4 and Il-10 levels were increased on the modSLA surface when compared to SLA surface (p<0.05). | **24-hours:** IL-1β, TNF-α and IL-6 were significantly (p<0.05) reduced on modSLA surfaces when compared to SLA on titanium and titanium-zirconia. IL-4 and IL-10 was found at significantly (p<0.05) increased levels for modSLA compared to SLA on titanium and titanium-zirconia.  **72-hours:** IL-1β, IL-6 and TNF-α on titanium modSLA surfaces were significantly (p<0.05) reduced compared to SLA. IL-1β on titanium-zirconia did not reach the level of statistical significance. IL-4 and IL-10 were significantly increased for modSLA compared to SLA on titanium and titanium-zirconium materials. | There was an overall trend for lower levels of pro-inflammatory cytokines IL-1β, IL-6 and TNF-α on the SLActive surface compared to the SLA along the measured timepoints. In addition, anti-inflammatory markers IL-4 and IL-10 were also seen to be reduced.  **24-hours:** For the overall time period there was no statistically significant difference found for IL-1β, IL-6 and TNF-α produced by macrophages on SLActive compared to SLA surfaces. In addition, IL-4 and IL-10 were significantly increased on SLActive surfaces compared to SLA surfaces (p<0.05).  . | **48-hours:** TNF-α, IL-1β, IL-6 and IL-12 levels were lower on titanium-zirconium with SLActive when compared to SLA. However, this did not reach statistical significance.  Anti-inflammatory cytokines IL-4 and IL-10 were found to be released in significantly (p<0.05) increased amounts for SLActive compared to SLA. | Not assessed | **48-hours:** SLActive titanium and titanium-zirconium surfaces had significantly reduced IL-1β, IL-6 and TNF-α when compared to SLA (p<0.05).  Titanium and titanium-zirconium SLActive surfaces had significantly (p<0.05) increased anti-inflammatory proteins IL-4 and IL-10 levels present in comparison to SLA. |

| **(…continued)** | **Hamlet *et al* (2012)** | **Alfarsi *et al* (2014)** | **Hotchkiss *et al* (2016)** | **Hotchkiss *et al* (2017)** | **Hotchkiss *et al* (2018)** | **Hotchkiss *et al* (2019)** | **Wang *et al* (2019)** | **Abaricia *et al* (2021b)** |
| --- | --- | --- | --- | --- | --- | --- | --- | --- |
| **Conclusions:** | This study found an overall downregulation of pro-inflammatory cytokine gene expression significantly for TNF-α and IL-1β. This may be in keeping with a phenotypic switch of macrophage towards the more reparative M2 phenotype. | The modulation of the pro-inflammatory macrophage response  by the modSLA surface treatment on grade II commercially pure titanium discs may result in a more reparative environment when compared to SLA surface treatment. | modSLA surface treatments stimulates more anti-inflammatory cytokine release by macrophages than on SLA surfaces.  In addition, there is a significant reduction of pro-inflammatory markers on modSLA surfaces when compared to SLA.  This shows that modSLA surfaces may alter the macrophage response from an M1 to an M2 phenotype which may lead to differing effects on the wound healing cascade.  . | The macrophage response to modSLA compared to SLA showed a reduced production of pro-inflammatory markers on titanium and titanium-zirconium materials.  In addition, the expression and release of anti-inflammatory markers occurred for modSLA surfaces when compared to SLA surfaces.  These results may indicate an immunomodulatory effect of modSLA surface treatment on macrophages towards an M2 anti-inflammatory phenotype compared to SLA. | This study that a hydrophilic SLA titanium surface can reduce pro-inflammatory and increase anti-inflammatory macrophage markers when compared to its hydrophobic counterpart. This shows that the surface modification of titanium implants can polarize macrophages towards a pro-wound healing, phenotype with increasing wettability. | SLActive surfaces upregulated and increased secretion of anti-inflammatory markers. Pro-inflammatory markers were decreased on SLActive surfaces; however, they did not reach statistical significance.  This study found that dental implants may be used as a rapid screening tool for the potential.  In addition, the differences in protein release and microenvironment contribute towards decreased healing and osseointegration times. | This study showed that modSLA surfaces are capable reducing of pro-inflammatory TNF-α and IL-6 gene expression. Furthermore, anti-inflammatory IL-10 levels were significantly increased on modSLA surfaces when compared SLA.  The enhanced hydrophility of the modSLA surface when compared to the hydrophobic SLA surfaces appears to modulate macrophage polarization. | This study found that surface hydrophilicity, mean roughness or chemical composition can alter macrophage phenotype, any of these along may be insufficient to predict modulation of the macrophage phenotype.  However, titanium and titanium-zirconium with SLActive surface treatment had a reduced pro-inflammatory response along with an increased production of anti-inflammatory markers in when compared to SLA. |
| **Does this study address the review question?** | Yes | Yes | Yes | Yes | Yes | Yes | Yes | Yes |
| **Source of funding** | Partial funding by Straumann AG, Basel, Switzerland. | King Khalid University and Griffith University co-funded this study. | Funded by the International Association of Dental Research-Academy of Osseointegration Innovation  in Implant Sciences Award. | Funding was by an Academy of Osseointegration Innovation in Implant Sciences Award, conducted by the International Association for Dental  Research and sponsored by the Academy of  Osseointegration.  Surfaces were provided by Institut Straumann AG, Basel, Switzerland. | Funding was through an Academy of Osseointegration Innovation in Implant Sciences Award, conducted by the International Association for Dental Research and sponsored by the Academy of Osseointegration.  Surfaces were provided by Institut Straumann AG, Basel, Switzerland. | Source of funding not stated. | Funded by the International Team of Implantology (ITI) Foundation grant. | Funding provided by the National Institutes of Health. |
| **Conflicts of interest statement:** | Statement not present | Statement not present | Statement not present | Statement not present | Statement not present | Statement not present | No conflicts of interests declared | Statement not present |
