# Supplementary material for: A systematic review comparing the macrophage inflammatory response to hydrophobic and hydrophilic sandblasted large grit, acid‐etched titanium or titanium–zirconium surfaces during in vitro studies
Source: Clin Exp Dent Res. 2023 Mar 29;9(3):437–48. doi: 10.1002/cre2.730 (PMC10280619; doi:10.1002/cre2.730)
Supplement: Supplementary file 5 — Supplementary information. [file CRE2-9-437-s005.docx]

Appendix 5: Outline of the assessment of quality of included studies.

| **Study Reference** | **Hamlet *et al.* (2012)** | **Alfarsi *et al.* (2014)** | **Hotchkiss *et al.* (2016)** | **Hotchkiss *et al.* (2017)** | **Hotchkiss *et al.* (2018)** | **Hotchkiss *et al.* (2019)** | **Wang *et al.* (2019)** | **Abaricia *et al.* (2021b)** |
| --- | --- | --- | --- | --- | --- | --- | --- | --- |
| **Materials** |  |  |  |  |  |  |  |  |
| Is the quality of the test material clearly stated? | **Yes** | **Yes** | **Yes** | **Yes** | **Yes** | **Yes** | **Yes** | **Yes** |
| Is the quality of the test material sufficient? | **Yes** | **Yes** | **Yes** | **Unclear** | **Yes** | **Yes** | **Yes** | **Yes** |
| Is it clear how the test material was manufactured? | **Yes** | **Yes** | **Yes** | **Yes** | **Yes** | **Yes** | **Yes** | **Yes** |
| Is the makeup of the comparator material clearly stated? | **Yes** | **Yes** | **Yes** | **Yes** | **Yes** | **Yes** | **Yes** | **Yes** |
| Is the makeup of the comparator material appropriate? | **Yes** | **Yes** | **Yes** | **Yes** | **Yes** | **Yes** | **Yes** | **Yes** |
| Was surface hydrophilicity evaluated? | **No** | **No** | **Yes** | **Yes** | **Yes** | **Yes** | **No** | **Yes** |
| Was surface roughness evaluated | **Yes** | **Yes** | **Yes** | **Yes** | **Yes** | **Yes** | **No** | **Yes** |
| Is the source of macrophage clearly stated? | **Yes - RAW 264.7 murine-derived macrophage cell line** | **Yes – Human acute monocytic leukemia cell-line THP-1** | **Yes – 6–8-week-old male C57BL/6 mice** | **Yes – 6–8-week-old male C57BL/6 mice** | **Yes – 8–12-week-old male C57BL/6 mice** | **Yes – 10–12-week-old male C57BL/6 mice** | **Yes - RAW 264.7 murine-derived macrophage cell line** | **Yes – purchased Human peripheral blood monocytes** |
| **Design** |  |  |  |  |  |  |  |  |
| Was macrophage differentiation performed appropriately? | **-** | **Unclear** | **Yes** | **Yes** | **Yes** | **Yes** | **-** | **Unclear** |
| Is it clear which factors are being studied? | **Yes** | **Yes** | **Yes** | **Yes** | **Yes** | **Yes** | **Yes** | **Yes** |
| Was there both an experimental and comparator group? | **Yes** | **Yes** | **Yes** | **Yes** | **Yes** | **Yes** | **Yes** | **Yes** |
| Was outcome assessment appropriate? | **Yes** | **Yes** | **Yes** | **Yes** | **Yes** | **Yes** | **Yes** | **Yes** |
| Were technical replicates performed? | **Yes** | **Yes** | **Yes** | **Yes** | **Yes** | **Yes** | **Yes** | **Yes** |
| Were experimental replicates performed? | **Yes** | **No** | **Yes** | **Yes** | **No** | **Yes** | **Yes** | **Yes** |
| Was measurement consistent across experimental groups? | **Yes** | **Yes** | **Yes** | **Yes** | **Yes** | **Yes** | **Yes** | **Yes** |
| Was measurement consistent for repeated experiments? | **Yes** | **Yes** | **Yes** | **Yes** | **Yes** | **Yes** | **Yes** | **Yes** |
| Were test methods well-established? | **Yes** | **Yes** | **Yes** | **Yes** | **Yes** | **Yes** | **Yes** | **Yes** |
| Was the outcome measured directly? | **Yes** | **Yes** | **Yes** | **Yes** | **Yes** | **Yes** | **Yes** | **Yes** |
| Was the outcome measured objectively? | **Yes** | **Yes** | **Yes** | **Yes** | **Yes** | **Yes** | **Yes** | **Yes** |
| Is it clear who undertook key parts of the experiment? | **No** | **No** | **No** | **No** | **No** | **Yes** | **No** | **Yes** |
| Were assessors blinded as to the surface treatment? | **Unclear** | **Unclear** | **Unclear** | **Unclear** | **Unclear** | **Unclear** | **Unclear** | **Unclear** |
| **(…continued)** | **Hamlet *et al.* (2012)** | **Alfarsi *et al.* (2014)** | **Hotchkiss *et al.* (2016)** | **Hotchkiss *et al.* (2017)** | **Hotchkiss *et al.* (2018)** | **Hotchkiss *et al.* (2019)** | **Wang *et al.* (2019)** | **Abaricia *et al.* (2021b)** |
| **Analysis** |  |  |  |  |  |  |  |  |
| Was sample loss at key points described? | **No** | **No** | **No** | **No** | **No** | **No** | **No** | **No** |
| Were appropriate statistical tests used? (parametric/non-parametric) | **Yes – significance was assessed with ANOVA. Pair-wise comparisons were performed using Tukey post-hoc testing.**  **Normality of data not assessed.** | **Unclear – Normality of data was not assessed.**  **Students t-test for gene expression pairwise comparison.**  **Statistical methods were not outlined for protein quantification analysis.** | **Yes – one-factor equal-variance ANOVA and post-hoc testing using the Tukey-HSD method.**  **However, normality of data not assessed.** | **Yes – QQ plot used to assess data normality**  **One-factor, equal-variance ANOVA used with Tukey HSD post-hoc testing** | **Yes – QQ plot used to assess data normality**  **One-factor, equal-variance ANOVA used with Tukey HSD post-hoc testing** | **Yes – QQ plots used to assess data normality**  **ANOVA used with TUKEY-HSD.** | **Yes – one- and two-way ANOVA with a Bonferroni test.**    **However, normality of data was not assessed.** | **Yes**  **Shapiro-Wilk was used to test for data normality.**  **A one-factor, equal ANOVA was used followed by post-hoc TUKEY-HSD for multiple comparisons** |
| Does data presented align with analyses conducted? | **Yes** | **Unclear – Data for Day 1 for gene expression is not presented.** | **Unclear - Data presented is from one experiment.** | **Unclear - Data presented is from one experiment.** | **Yes** | **Unclear - Data presented is from one experiment** | **Unclear – Data for Day 5 is not displayed or discussed** | **Yes** |
| Is variability within each condition reported? | **Yes** | **No** | **Yes** | **Yes** | **Yes** | **Yes** | **No** | **Yes** |
|  |  |  |  |  |  |  |  |  |
| **Funding** |  |  |  |  |  |  |  |  |
| May the source of funding have invested interests in the outcomes? | **Yes** | **No** | **No** | **No** | **No** | **Unclear source of funding.** | **No** | **No** |
| Were materials supplied by parties with possible invested interests? | **Yes** | **Yes** | **Yes** | **Yes** | **Yes** | **Yes** | **Yes** | **Yes** |
|  |  |  |  |  |  |  |  |  |
| **Relevance** |  |  |  |  |  |  |  |  |
| Are the test conditions clinically relevant? | **No** | **No** | **No** | **No** | **No** | **No** | **No** | **No** |
